# Epidemiological insights and healthcare challenges of tuberous sclerosis complex in Shizuoka Prefecture: a retrospective cohort study

**DOI:** 10.1186/s13023-025-03799-w

**Published:** 2025-05-23

**Authors:** Satoshi Kishida, Eiji Nakatani, Takeshi Usui, Shuhei Fujimoto, Seiichiro Yamamoto, Yoshiki Miyachi

**Affiliations:** 1 Graduate School of Public Health, Shizuoka Graduate University of Public Health, 4-27-2 Kitaando, Aoi-ku, Shizuoka, 420-0881 Japan; 2 Department of Biostatistics and Health Data Science, Graduate School of Medical Science Nagoya City University, Nagoya, Japan

**Keywords:** Tuberous sclerosis complex, Epidemiology, Prevalence, Shizuoka Prefecture, Mammalian target rapamycin inhibitors, Diagnostic strategies

## Abstract

**Background:**

Tuberous sclerosis complex (TSC) is a rare genetic disorder characterized by benign tumors in multiple organs, leading to significant morbidity. Despite its recognition as a rare disease in Japan, comprehensive regional epidemiological data are lacking, hindering effective healthcare resource allocation and the development of targeted therapies. This study aimed to determine the prevalence and epidemiological characteristics of TSC in Shizuoka Prefecture and assess the associated healthcare burden.

**Methods:**

We conducted a retrospective cohort study using data from the Shizuoka Kokuho Database, covering approximately 2.4 million residents over an 8.5-year period (April 2012–September 2020). TSC cases were identified using the International Classification of Diseases, 10th Revision (ICD-10) code Q85.1. Adjusted prevalence rates were calculated, and analyses were performed on patient demographics, healthcare utilization, comorbidities, and treatment patterns. Comparisons with the general population were made to assess differences in neurological and renal complications.

**Results:**

A total of 125 patients diagnosed with TSC were identified, resulting in an adjusted prevalence rate of 10.2 per 100,000. Diagnosis rates increased after 2012, likely due to revised diagnostic criteria and the introduction of mammalian target of rapamycin (mTOR) inhibitors. Patients with TSC exhibited substantial healthcare utilization and significantly higher rates of neurological and renal complications compared to the general population. Disease prevalence was notably higher among young males and varied across different age groups.

**Conclusions:**

The higher prevalence of TSC observed in Shizuoka Prefecture suggests potential underestimation in other regions. The findings underscore the need for enhanced diagnostic strategies, including widespread genetic testing and updated treatment protocols, to effectively manage the diverse manifestations of TSC. Continuous epidemiological monitoring and adaptive healthcare policies are essential to improve timely diagnosis and the overall quality of life for patients with TSC. This study supports the necessity of patient-centered approaches in managing chronic rare diseases.

**Supplementary Information:**

The online version contains supplementary material available at 10.1186/s13023-025-03799-w.

## Introduction

Tuberous sclerosis complex (TSC), a genetic disorder characterized by the development of benign tumors across various body organs, significantly impacts patient health. Designated as a rare disease in Japan in 2015 [1], TSC has an estimated global prevalence of approximately 1 in 6,000 individuals [2, 3], suggesting that 15,000 individuals in Japan are potentially affected. However, accurate patient numbers remain elusive [1], marking a substantial gap in the comprehensive understanding and management of TSC.

The management of rare diseases such as TSC is fraught with challenges [4], notably the often protracted time from symptom onset to diagnosis [5]. Addressing this issue, the International Consortium for Rare Diseases emphasizes the importance of diagnosing the disease and beginning treatment within 1 year of symptom presentation [6]. The diagnostic journey for TSC is intricate and requires the careful evaluation of symptoms, which span multiple organs including the skin, nervous system, kidneys, liver, lungs, gastrointestinal tract, bones, eyes, and teeth [7]. Age-related variations in symptom presentation and individual differences add layers of complexity to the diagnosis and effective treatment of TSC [8, 9].

After diagnosis, ongoing management is guided by the International TSC Conference Group, which recommends regular assessments tailored to the patient’s specific symptoms [10]. The limited number of specialized treatment facilities often forces patients to travel considerable distances, further exacerbating patients’ burden. Additionally, mammalian target rapamycin (mTOR) inhibitors have improved treatment outcomes but have not provided a cure and necessitate continuous treatment and monitoring [10].

This study aimed to accurately determine the prevalence and epidemiological characteristics of TSC in Shizuoka Prefecture. By focusing on the challenges encountered before diagnosis and the ongoing needs after diagnosis, we highlighted areas where strategic interventions can significantly alleviate the disease’s impact. Through this approach, we sought to enhance our understanding of TSC from the perspective of the patient journey to better inform policy and healthcare strategies, thus improving outcomes for individuals who live with this complex condition.

## Methods

### Data source

This study used the Shizuoka Kokuho Database, a comprehensive dataset that is linked to the Federation of National Health Insurance Association subscribers. The database includes a wide range of information, such as demographic details, registration data, medical claims, and health checkup records for individuals who reside in Shizuoka Prefecture [11]. With a total population of approximately 3.6 million, Shizuoka Prefecture is representative of Japan’s climatic conditions and population distribution. The database captures data from 25% of residents under 65 years old and 75% of those aged 65 and above. The SKDB primarily includes data from NHI and LSEMCS, which together cover all elderly residents over 75 years and a substantial portion of those under 75 years. However, it does not include individuals covered by employer-based insurance or those who receive healthcare exclusively through private services. Japan has a universal healthcare system, and cases of uninsured patients or individuals relying entirely on private healthcare are exceedingly rare.

### Study design, data availability period, and study population

**Fig. 1:**
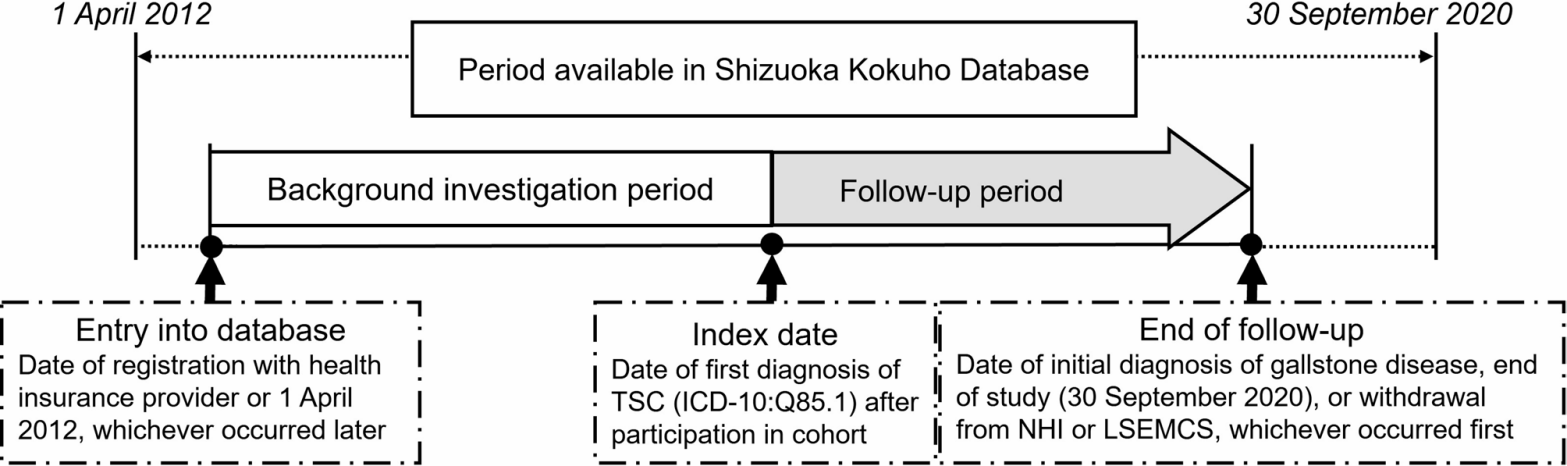
Study Schema. “Cohort entry” is defined as the date of registration with the health insurance provider or 1 April 2012, whichever occurred later. “Index date” is defined as the date of first diagnosis of TSC (ICD-10: Q85.1) after cohort participation. “Follow-up period” is defined as the interval between the index date and (1) the end of the study (30 September 2020), (2) the withdrawal date from the health insurance system, or (3) the date of death, whichever occurred first. ICD-10: International Classification of Diseases − 10th Revision, TSC: tuberous sclerosis complex, NHI: National Health Insurance, LSEMCS: Late-Stage Elderly Medical Care System

The retrospective cohort study used the comprehensive Shizuoka Kokuho Database. Detailed schematics of the study design are depicted in Fig. 1, which provides a visual representation of the data collection methodology. This approach facilitated a thorough analysis of longitudinal data that covered 8.5 years from April 2012 through September 2020 (SKDB2021 version).

Data for each participant were available based on their active enrollment in the insurance database. The period extended from the later of two starting points—the date of the participant’s initial registration with the insurance scheme or April 2012—and ended on the earlier of two endpoints—the date of the participant’s withdrawal from insurance or September 2020. In addition, the database captures all deaths occurring during the study period, including those among patients diagnosed with TSC.

The study population comprised all individuals in the database for whom linked data regarding annual health checkups and insurance claims was available. This setup ensured the inclusion of comprehensive health records for each participant throughout the study period.

### Identification of tuberous sclerosis complex cases

Patients were identified as having TSC from insurance claims that displayed the International Classification of Diseases − 10th Revision (ICD-10) code Q85.1, which is specific to the condition [12].

### Statistical analysis

Continuous variables were reported as means and standard deviations, whereas categorical variables were summarized as frequencies and percentages. Basic descriptive statistics, including prevalence rates, sex, age distributions, treatment patterns, and comorbidity prevalence, were used to assess TSC patient characteristics. Data on medical interventions, such as surgical treatments and prescriptions of mTOR inhibitors, were cataloged and detailed in specific tables. We incorporated Pearson’s chi-square test to assess differences in prevalence rates between males and females, as well as among different age groups within each sex. Additionally, Pearson’s chi-square test was performed to evaluate differences in the prevalence of comorbid conditions between TSC patients and the non-TSC population. This analysis aimed to determine whether significant disparities exist in the occurrence rates of epilepsy, psychotic and affective disorders, and renal complications between the two groups. A simple missing data imputation was not conducted. All analyses were conducted using JMP Pro17.0.0 software (SAS Institute, Cary, NC, USA).

## Results

### Prevalence of tuberous sclerosis complex by sex and age in 2019

**Fig. 2:**
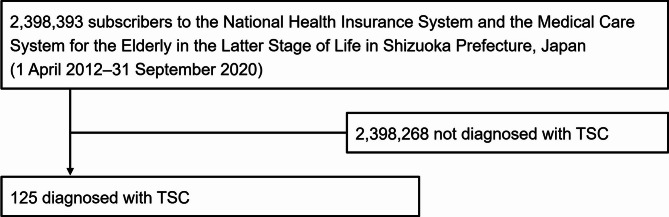
Flow chart diagram. TSC: tuberous sclerosis complex

In an analysis of the Shizuoka Kokuho Database, which includes data on 2,398,393 individuals, we specifically examined the records of 125 patients diagnosed with TSC (Fig. 2). The total number of individuals (2,398,393) refers to all persons registered in SKDB between 2012 and 2020. In contrast, the number of individuals in 2019 (1,401,399) includes only those covered for at least one day that year.

Among 1,401,399 registrants in 2019, 85 were identified as having TSC. Therefore, the initial prevalence rate was estimated at 6.1 per 100,000. After adjusting for the age-specific demographics of Shizuoka Prefecture, the rate increased to 10.2 per 100,000. The sex distribution among the patients showed minimal variance (41 men [48.2%] and 44 women [51.8%]). The median age on 1 January 2019 was 44.0 years, with a range from 1 to 89 years (42 years for men and 44 years for women), indicating a balanced occurrence of TSC across the sexes. There was no significant difference in prevalence between male and female (*P* = 0.562), whereas significant differences were observed in the age-specific prevalence in male (*P* < 0.001) and female (*P* < 0.001). Detailed prevalence data segmented by sex and age are presented in Table 1. Our analysis highlights that TSC prevalence varies significantly across groups; the highest rates are observed in young men aged 0–19 years, and the lowest rates are in individuals aged 65 years and older.

**Table 1:** Prevalence of patients with TSC by sex and age

Group	Number of patients with TSC	Population	Prevalence (per 100,000)	95% confidence interval
Total	85	1,401,314	6.07	-
**Male**	41	632,033	6.49	4.78–8.80
Age 0–19 years	10	54,680	18.29	9.93–33.70
Age 20–64 years	24	281,428	8.53	5.73–12.70
Age 65 + years	7	295,925	2.37	1.15–4.88
**Female**	44	769,281	5.72	4.26–7.68
Age 0–19 years	8	52,004	15.38	7.80–30.40
Age 20–64 years	27	315,499	8.56	5.88–12.50
Age 65 + years	9	401,778	2.24	1.18–4.26

TSC: tuberous sclerosis complex. There was no significant difference in prevalence between male and female (*P* = 0.562), whereas significant differences were observed in the age-specific prevalence in male (*P* < 0.001) and female (*P* < 0.001)

### Characteristics and outcomes of patients with TSC classified by age at diagnosis

In our study, 125 patients diagnosed with TSC were analyzed based on age at diagnosis. The distribution of patients was as follows: 23.2% were under the age of 20 years, 60.8% fell within the 20 to 64-year age range, and 16.0% were over 65 years of age. The median age at diagnosis varied significantly across these groups, with those under 20 years diagnosed at a median age of 5.0 years, those between 20 and 64 years at 38.0 years, and those over 65 years at 74.5 years. The overall median age at diagnosis across all age groups was 44.0 years. Detailed patient characteristics including sex distribution and the prevalence of various comorbid conditions are detailed in Table 2. Over the average observation period of 65.7 months, 12 deaths were recorded; deaths were split evenly, with six occurring in each of the 20–64-year and over 65-year age groups.

**Table 2:** Characteristics of all and age group-specific patients with TSC

Variable (unit)	Category	Age group at diagnosis in years			Total
		0–19	20–64	65+	
		(*n* = 29) 23.2%	(*n* = 76) 60.8%	(*n* = 20) 16.0%	(*n* = 125)
Median age in years		5.0	38.0	74.5	44.0
Sex (%)	Male	15 (51.7)	35 (46.0)	9 (45.0)	59 (47.2)
**Comorbid conditions**	Yes				
Hemangiomyolipoma (%)	Yes	0	2 (2.6)	0	2 (1.6)
Cardiac rhabdomyosarcoma (%)	Yes	1 (3.5)	0	0	1 (0.8)
Developmental and cognitive disorders (%)	Yes	1 (3.5)	4 (5.3)	0	5 (4.0)
Epilepsy (%)	Yes	18 (62.1)	47 (61.8)	3 (15.0)	68 (54.4)
Lung lymphangioleiomyomatosis (%)	Yes	0	4 (5.26)	1 (5.0)	5 (4.0)
Polycystic kidney disease (%)	Yes	0	1 (1.3)	0	1 (0.8)
Psychotic and affective disorders (%)	Yes	9 (31.0)	31 (40.8)	7 (35.0)	47 (37.6)
Renal angiomyolipoma (%)	Yes	0	20 (26.3)	1 (5.0)	21 (16.8)
Renal cancer (%)	Yes	0	2 (2.6)	3 (15.0)	5 (4.0)
Renal cysts (%)	Yes	0	3 (4.0)	2 (10.0)	5 (4.0)
Retinal tumors (%)	Yes	0	0	0	0
Subependymal giant cell astrocytoma (%)	Yes	0	1 (1.3)	0	1 (0.8)
Angiofibroma (%)	Yes	0	2 (2.6)	0	2 (1.6)
Leukoplakia (%)	Yes	0	0	0	0
Neoplastic lesion (benign; %)	Yes	1 (3.5)	30 (39.5)	4 (20.0)	35 (28.0)
Neoplastic lesion (unknown; %)	Yes	11 (37.9)	31 (40.8)	5 (25.0)	47 (37.6)

TSC: tuberous sclerosis complex

Continuous variables are presented as median (range), and categorical variables are presented as frequency (%)

### Surgical and Pharmacological management of patients with TSC

Our study analyzed the medical interventions in 125 patients with TSC and categorized patients by age group (Table 3). A noTable 39.2% of patients underwent surgical procedures, whereas 25.6% received prescriptions for mTOR inhibitors. The most common surgical sites were skin or soft tissue (18.4%), followed by unknown sites (10.4%) and the digestive system (4.8%). Surgeries involving the head or neck (2.4%), renal system (2.4%), and lungs (0.8%) were less frequent. Other post-diagnosis testing in these patients is provided in Supplementary Table 1.

**Table 3:** Surgical treatment and treatment with mTOR inhibitors for all and age group-specific patients with TSC

Treatment	Age group at diagnosis in years			Total
	0–19	20–64	65+	
	(*n* = 29)	(*n* = 76)	(*n* = 20)	(*n* = 125)
Surgical treatment	5 (17.2)	35 (46.1)	9 (45.0)	49 (39.2)
Head or Neck	0	3 (3.9)	0	3 (2.4)
Lung	0	0	1 (5.0)	1 (0.8)
Renal	0	3 (3.9)	0	3 (2.4)
Digestive System	0	1 (1.3)	5 (25.0)	6 (4.8)
Skin or Soft Tissue	3 (10.3)	17 (22.4)	3 (15.0)	23 (18.4)
Unknown Site	2 (6.9)	11 (14.5)	0	13 (10.4)
Treatment with mTOR inhibitor	6 (20.7)	22 (29.0)	4 (20.0)	32 (25.6)
Everolimus tablet	0	14 (18.4)	2 (10.0)	16 (12.8)
Everolimus dispersion tablet	4 (13.8)	1 (1.3)	0	5 (4.0)
Sirolimus tablet	0	1 (1.3)	0	1 (0.8)
Sirolimus gel	4 (13.8)	9 (11.8)	0	13 (10.4)

TSC: tuberous sclerosis complex, mTOR: mammalian target rapamycin

### Comparison of comorbidity profiles between patients with TSC and the general population

In this study, we assessed the prevalence of comorbid conditions among 125 patients diagnosed with TSC and compared these rates with those in a large cohort of 2,398,268 individuals without TSC. The analysis revealed that patients with TSC are significantly more likely than the broader population to suffer from a range of comorbidities including epilepsy, psychotic and affective disorders, and renal complications. Comprehensive statistics that detail these disparities are tabulated in Table 4. The tumor prevalence in individuals with and without TSC is provided in Supplementary Table 2. The comparison underscores the heightened health burden of those diagnosed with TSC.

**Table 4:** Prevalence of comorbidities characterized by TSC in individuals with and without TSC

Comorbidity	Patients with TSC	Individuals without TSC	*P*-value
	(*n* = 125)	(*n* = 2,398,268)	
Hemangiomyolipoma	3 (2.4)	43 (0.0)	< 0.001
Cardiac rhabdomyoma	2 (1.6)	0	< 0.001
Developmental and cognitive disorders	11 (8.8)	9,782 (0.4)	< 0.001
Epilepsy and status epilepticus	75 (60.0)	84,570 (3.5)	< 0.001
Lung lymphangioleiomyomatosis	10 (8.0)	21 (0.0)	< 0.001
Any Lung Condition	6 (4.8)	64,101 (2.7)	0.140
Polycystic kidney disease	4 (3.2)	2,205 (0.1)	< 0.001
Psychotic and affective disorders	59 (47.2)	536,432 (22.4)	< 0.001
Any Renal Condition	56 (44.8)	284,526 (11.9)	< 0.001
Renal angiomyolipoma	36 (28.8)	807 (0.0)	< 0.001
Malignant neoplasms of urinary tract	8 (6.4)	23,611 (1.0)	< 0.001
Cyst of kidney	8 (6.4)	68,381 (2.9)	0.017
Retina	0	5 (0.0)	0.987
Subependymal giant cell astrocytoma	5 (4.0)	0	< 0.001
Angiofibroma	5 (4.0)	3 (0.0)	< 0.001
Vitiligo	1 (0.8)	5,040 (0.2)	0.150

TSC: tuberous sclerosis complex

Hemangiomyolipoma (disease code: 8847010), cardiac rhabdomyoma (disease code: 2398126), developmental and cognitive disorders (F71-78, F84, F98), epilepsy and status epilepticus (ICD-10: G40, G41), lung lymphangioleiomyomatosis (disease code: 8843635), any lung condition (ICD-10: J80, J81, J82, J83, J84, J98), polycystic kidney disease (Q61), psychotic and affective disorders (F06–07, F09, F20–29, F30–39, F40–48, F60–69, F70–79, F80–89, F90–98), any renal condition (ICD-10: C64, D17, D30, I12-13, N10–19, N281), renal angiomyolipoma (disease code: 8844065, 8835603, 8847971), malignant neoplasms of urinary tract (ICD-10: C64–68), cyst of kidney (ICD-10: N281), retina (ICD-10: D312), subependymal giant cell astrocytoma (disease code: 8847493), angiofibroma (disease code: 2159049, 8833091), vitiligo (ICD-10: L80)

## Discussion

This comprehensive retrospective cohort study uses extensive data from the Shizuoka Kokuho Database and provides a detailed epidemiological and clinical profile of TSC in a significant segment of the Japanese population. We identified critical aspects of TSC management, including the prevalence and demographic distribution of the disease, revealing an adjusted prevalence of 10.2 per 100,000 in 2019. Our findings underscore a variable disease impact across age groups, with the highest prevalence among young men aged 0–19 years. Additionally, the management strategies involving surgical and pharmacological treatments indicate substantial utilization of healthcare resources, particularly the significant use of mTOR inhibitors such as everolimus and sirolimus. The comparison of comorbidity profiles further highlights the increased health burden experienced by patients with TSC, particularly with regard to conditions such as epilepsy, psychotic and affective disorders, and renal complications. This study lays the groundwork for addressing gaps in care and tailoring interventions to improve the quality of life and outcomes for patients with TSC. Additionally, some surgical interventions and off-label medication uses might not be captured if patients received them outside the NHI/LSEMCS framework. However, as this study is based on health insurance claims data, we could not determine the specific indications for surgery or mTOR inhibitor use. Instead, we have specified the anatomical sites of surgery in Table 3. Surgical treatments may have been applied to various TSC-related conditions affecting the head and neck, lungs, kidneys, and skin. Similarly, while mTOR inhibitors were prescribed for TSC, their precise treatment purposes remain unclear. We acknowledge these limitations and note that future studies using hospital or registry data are needed to clarify treatment indications.

The observed prevalence of TSC in this study (10.2 per 100,000) significantly exceeds previously reported rates both Japanese and global rates, which generally range from 3.9 to 7.9 per 100,000 [13, 14, 15, 16]. This notable discrepancy can be attributed to several factors. Chief among the factors is the 2012 revision by the International TSC Conference Group of the TSC diagnostic criteria, which were expanded to include genetic testing and more detailed assessments of patient and family medical histories—even in patients who lack overt clinical symptoms [1, 10]. This expansion of diagnostic criteria is likely a major contributor to the increased diagnosis rate observed because it allows for earlier and more accurate identification of the condition. Such changes reflect a significant shift toward a more inclusive and sensitive approach to diagnosing TSC and potentially capture a higher number of mild or atypical patients who previously went unrecognized. The differences in prevalence across age groups may also reflect increased genetic testing and greater awareness of TSC, leading to higher diagnosis rates in younger individuals. Conversely, older individuals may include previously undiagnosed cases, particularly those with mild or asymptomatic forms. For patients diagnosed at 65 years or older, diagnostic challenges may have contributed to delayed detection. TSC diagnosis requires assessment across multiple specialties [1], and variability in symptom onset, severity, and presence further complicates identification [8]. Additionally, patients with intellectual disabilities or neuropsychiatric symptoms may struggle to communicate their condition accurately, increasing the risk of missed diagnosis [22]. These factors likely result in TSC remaining undiagnosed in early life and only being recognized in older patients when complications arise.

The expanded availability of mTOR inhibitors such as everolimus and sirolimus has significantly broadened treatment horizons. Initially approved in 2012 for the treatment of renal angiomyolipoma and giant cell astrocytoma associated with TSC, everolimus had its indications extended in 2020 to encompass a broader range of TSC manifestations [17, 18]. Similarly, sirolimus was approved in 2012 for pulmonary lymphangioleiomyomatosis and later, in 2018, for TSC-related skin diseases [19, 20]. The introduction of these targeted therapies likely played a role in promoting more rigorous screening and diagnostic practices. This hypothesis is supported by other studies in Japan that demonstrate a correlation between the approval of such drugs and an increase in the identification of patients with TSC [21]. The heightened awareness and ability to manage symptoms pharmacologically may have led to increased diagnosis as clinicians and patients alike became more vigilant in recognizing the signs of TSC.

The 2015 classification of TSC as an intractable disease in Japan and the enhanced healthcare benefits and financial subsidies provided under this designation likely contributed significantly to the increase in diagnoses. This policy change is reflected in the increase in the prevalence rate from 5.4 per 100,000 in 2014 to 6.1 per 100,000 in 2015, marking the most substantial annual increase observed during the study period. The designation not only improved access to specialized healthcare services and support for patients with TSC but also likely increased awareness among healthcare providers and patients, enhancing vigilance in identifying and diagnosing TSC at earlier stages.

Although our methodology for identifying patients with TSC boasts high specificity, the reported prevalence rates may nevertheless not fully represent the actual scope of the condition. Future studies integrating multiple insurance datasets would provide a more comprehensive view of TSC healthcare utilization in Japan. This potential underrepresentation could stem from historical cases that were diagnosed before our study period and patients who had not recently engaged with hospital services. Additionally, the integration of genetic testing into insurance coverage starting in 2021 is expected to significantly enhance the accuracy of TSC diagnoses. This advancement underscores the importance of ongoing surveillance to accurately track and elucidate the evolving epidemiology of TSC.

### Limitations

This study predominantly relies on retrospective data, which inherently limits our ability to track the long-term progression of TSC. A significant concern is that older patients might have remained undiagnosed given inadequate screening methods available during their younger years. Moreover, dependence on administrative data restricted our ability to comprehensively assess patient-reported outcomes and detailed symptom development, potentially skewing our understanding of the disease’s impact. In particular, symptom data were collected only from physician-recorded diagnoses, meaning that not all clinically present symptoms were captured. This may have led to an underestimation of symptom prevalence. Due to this limitation, we did not perform statistical comparisons of symptom prevalence across age groups and instead presented descriptive statistics to highlight potential trends. Additionally, the database used in this study did not capture genetic testing results, as TSC genetic testing was not covered by Japan’s national health insurance during the study period, making data on its implementation unavailable. Another limitation is the potential selection bias inherent in using a single regional database, which might not capture the diversity of TSC cases nationwide. Patients obtaining surgical interventions or medications through private payment or employer-based insurance may not be represented. This could result in an underestimation of healthcare utilization among TSC patients. Additionally, the changes in healthcare policy and diagnostic criteria over time, which can affect the diagnosis rates and recorded prevalence independently of the actual disease spread, may have influenced the study results. Future research incorporating broader insurance data sources would enhance the comprehensiveness of our findings.

## Conclusions

This investigation into the prevalence and epidemiological characteristics of TSC in Shizuoka Prefecture provided valuable insights into the condition. The observed prevalence rate, which is higher than that reported in previous studies, suggests that changes in diagnostic criteria, advancements in treatment options, and updates to policy may have influenced these figures. These findings highlight the dynamic nature of epidemiological surveillance and the need for healthcare strategies that can adapt to evolving diagnostic and treatment landscapes. Although this study enhances our understanding of TSC in Shizuoka, it also underscores the importance of ongoing research to continually improve patient care protocols and outcomes for individuals who live with this challenging condition.

## Electronic supplementary material

Below is the link to the electronic supplementary material.

Supplementary Material 1

## Data Availability

According to Shizuoka Prefecture’s data use agreement with local insurers, the authors cannot provide the analyzed data to readers to protect the privacy of study participants. However, researchers interested in accessing this dataset may submit an application to Shizuoka Prefecture for restricted access. For further information, please contact the staff at Shizuoka Graduate University of Public Health (e-mail: info@s-sph.ac.jp).
