# Supplementary material for: Epidemiological insights and healthcare challenges of tuberous sclerosis complex in Shizuoka Prefecture: a retrospective cohort study
Source: Orphanet J Rare Dis. 2025 May 23;20:245. doi: 10.1186/s13023-025-03799-w (PMC12103018; doi:10.1186/s13023-025-03799-w)
Supplement: Supplementary file 1 — Supplementary Material 1 [file 13023_2025_3799_MOESM1_ESM.docx]

**Supplementary Table 1.** Post-diagnosis testing in all and age group-specific patients with TSC

| **Post-diagnosis testing performed** | **Age group at diagnosis (registration) in years** | | | **Total** |
| --- | --- | --- | --- | --- |
|  | **0–19** | **20–64** | **65+** |  |
| CT | 15 (51.7) | 56 (73.7) | 19 (95.0) | 90 (72.0) |
| MRI | 20 (69.0) | 39 (51.3) | 13 (65.0) | 72 (57.6) |
| ECG | 1 (3.5) | 2 (2.6) | 2 (10.0) | 5 (4.0) |
| Ultrasonography | 23 (79.3) | 49 (64.5) | 17 (19.1) | 89 (71.2) |

CT: computed tomography, MRI: magnetic resonance imaging, ECG: Electrocardiogram, TSC: tuberous sclerosis complex

**Supplementary Table 2.** Tumor prevalence in individuals with and without TSC

| **Tumor** | **Patients with TSC**  **(N=125)** | **Individual without TSC**  **(N=2,398,268)** |
| --- | --- | --- |
| **ICD-10, malignant neoplasms (%)** | 24 (19.2) | 287,711 (12.0) |
| C00–C14, malignant neoplasms of lip, oral cavity, and pharynx | 1 (0.8) | 7,120 (0.3) |
| C15–C26, malignant neoplasms of digestive organs | 3 (2.4) | 133,120 (5.6) |
| C30–C39, malignant neoplasms of respiratory and intrathoracic organs | 3 (2.4) | 41,866 (1.8) |
| C40–C41, malignant neoplasms of bone and articular cartilage | 0 (0.0) | 415 (0.0) |
| C43–C44, melanoma and other malignant neoplasms of skin,  C45–C49 malignant neoplasms of mesothelial and soft tissue | 2 (1.6) | 11,105 (0.5) |
| C50–C50, malignant neoplasm of breast | 1 (0.8) | 29,596 (1.2) |
| C51–C58, malignant neoplasms of female genital organs | 1 (0.8) | 13,410 (0.6) |
| C60–C63, malignant neoplasms of male genital organs | 4 (3.2) | 41,972 (1.8) |
| C64–C68, malignant neoplasms of urinary tract | 8 (6.4) | 23,611 (1.0) |
| C69–C72, malignant neoplasms of eye, brain, and other parts of central nervous system | 2 (1.6) | 1,662 (0.1) |
| C73–C75, malignant neoplasms of thyroid and other endocrine glands | 1 (0.8) | 6,061 (0.3) |
| C76–C80, malignant neoplasms of ill-defined, secondary, and unspecified sites | 9 (7.2) | 73,262 (3.1) |
| C81–C96, malignant neoplasms, stated or presumed to be primary, of lymphoid, hematopoietic, and related tissue | 1 (0.8) | 21,495 (0.9) |
| **ICD-10, in situ neoplasms and benign neoplasms (%)** | 51 (40.8) | 185,384 (7.7) |
| D00–D09, in situ neoplasms | 0 | 3,440 (0.1) |
| D10, benign neoplasm of mouth and pharynx  D11, benign neoplasm of major salivary glands | 0 | 1,555 (0.1) |
| D12, benign neoplasm of colon, rectum, anus, and anal canal  D13 benign neoplasm of other and ill-defined parts of digestive system | 0 | 34,938 (1.5) |
| D14, benign neoplasm of middle ear and respiratory system  D15, benign neoplasm of other and unspecified intrathoracic organs | 3 (2.4) | 2,005 (0.1) |
| D16, benign neoplasm of bone and articular cartilage | 0 | 1,663 (0.1) |
| D17, benign lipomatous neoplasm | 38 (30.4) | 12,026 (0.5) |
| D18, hemangioma and lymphangioma, any site | 6 (4.8) | 18,347 (0.8) |
| D19, benign neoplasm of mesothelial tissue  D20, benign neoplasm of soft tissue of retroperitoneum and peritoneum  D21, other benign neoplasms of connective and other soft tissue | 19 (15.2) | 2,904 (0.1) |
| D22, melanocytic naevi  D23, other benign neoplasms of skin | 9 (7.2) | 34,105 (1.4) |
| D24, benign neoplasm of breast | 0 | 1,663 (0.07) |
| D25, leiomyoma of uterus  D26, other benign neoplasms of uterus  D27, benign neoplasm of ovary  D28, benign neoplasm of other and unspecified female genital organs | 9 (7.2) | 67,903 (2.8) |
| D31, benign neoplasm of eye and adnexa,  D32, benign neoplasm of meninges,  D33, benign neoplasm of brain and other parts of central nervous system | 1 (0.8) | 5,962 (0.3) |
| D34, benign neoplasm of thyroid gland,  D35, benign neoplasm of other and unspecified endocrine glands | 0 | 6,682 (0.3) |
| D36, benign neoplasm of other and unspecified sites | 0 | 7,920 (0.3) |
| **ICD-10, neoplasms of uncertain or unknown behavior (%)** | 56 (44.8) | 234,669 (9.8) |
| D37, neoplasm of uncertain or unknown behavior of oral cavity and digestive organs | 3 (2.4) | 50,609 (2.1) |
| D38, neoplasm of uncertain or unknown behavior of middle ear and respiratory and intrathoracic organs | 2 (1.6) | 19,116 (0.8) |
| D39, neoplasm of uncertain or unknown behavior of female genital organs | 4 (3.2) | 17,235 (0.7) |
| D40, neoplasm of uncertain or unknown behavior of male genital organs | 0 | 1,415 (0.1) |
| D41, neoplasm of uncertain or unknown behavior of urinary organs | 21 (16.8) | 12,008 (0.5) |
| D42, neoplasm of uncertain or unknown behavior of meninges | 0 | 292 (0.0) |
| D43, neoplasm of uncertain or unknown behavior of brain and central nervous system | 15（12.0） | 11,308 (0.5) |
| D44, neoplasm of uncertain or unknown behavior of endocrine glands | 3 (2.4) | 34,608 (1.4) |
| D47, other neoplasms of uncertain or unknown behavior of lymphoid, hematopoietic, and related tissue | 0 | 2,464 (0.1) |
| D48, neoplasm of uncertain or unknown behavior of other and unspecified sites | 28 (22.4) | 115,836 (4.8) |

ICD-10: International Classification of Diseases - 10^th^ revision, TSC: tuberous sclerosis complex
